# A Rare Cause of Stroke in Young Adults: Occlusion of the Middle Cerebral Artery by a Meningioma Postpartum

**DOI:** 10.1155/2013/652538

**Published:** 2013-10-02

**Authors:** Stéphane Mathis, Benoît Bataille, Samy Boucebci, Marion Jeantet, Jonathan Ciron, Laurène Vandamme, Jean-Philippe Neau

**Affiliations:** ^1^Department of Neurology, CHU Poitiers, University of Poitiers, 2 rue de la Milétrie, 86021 Poitiers, France; ^2^Department of Neurosurgery, CHU Poitiers, University of Poitiers, 2 rue de la Milétrie, 86021 Poitiers, France; ^3^Department of Radiology, CHU Poitiers, University of Poitiers, 2 rue de la Milétrie, 86021 Poitiers, France; ^4^Department of Pathology, CHU Poitiers, University of Poitiers, 2 rue de la Milétrie, 86021 Poitiers, France

## Abstract

Meningioma is the most common nonglial intracranial primary tumor. It is a slowly growing tumor and presents clinically by causing seizures along with neurological or neuropsychological deficit. However, acute presentation of meningioma is possible. We are reporting a case of cerebral infarction due to a sphenoid wing meningothelial meningioma (with progesterone receptor positivity) leading to an occlusion of the middle cerebral artery (MCA) in a 30-year-old right-handed woman (1 month after childbirth). After surgery, no new neurological event occurred, and she recovered most of her neurological functions. Strokes due to meningioma are a highly rare clinical occurrence but should be given serious consideration, particularly in young patients.

## 1. Introduction

Meningioma is the most common nonglial intracranial primary tumor, accounting for 13 to 26% of all primary intracranial tumors [1]. This slowly growing tumor arises from the arachnoid cells of the leptomeninges and presents clinically by causing seizures along with neurological or neuropsychological deficit [2]. Meningiomas variably express hormone receptors for progesterone, androgen, estrogen, placenta growth factor, and exogenous hormones [3, 4] and are also 2-3 times more common in women than in men [5].

## 2. Case Report

**Figure 1:**
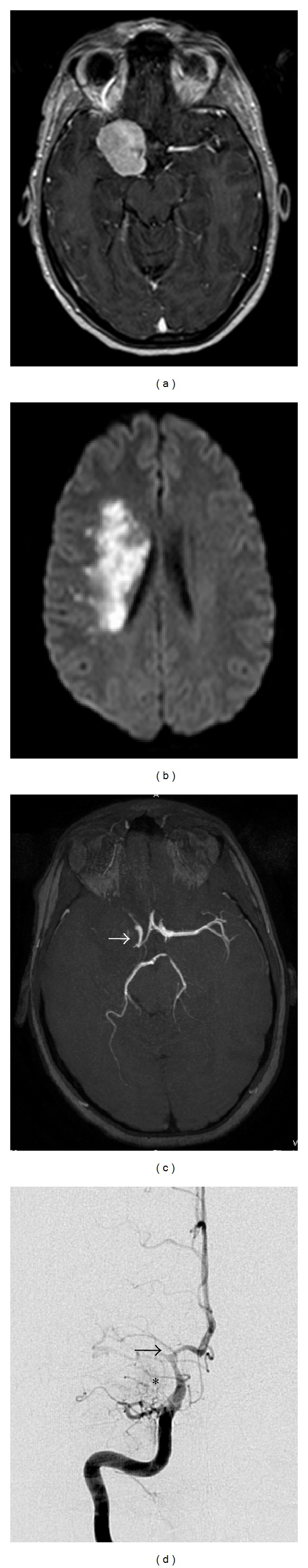
Brain MRI T1-weighted postgadolinium sequence showing homogenous enhancement of the meningioma (a). Brain MRI diffusion-weighted sequence showing acute deep MCA stroke (b). Brain MR angiography (axial sequence) showing occlusion of the right MCA (arrow) (c). Angiogram of the right internal carotid artery showing complete occlusion of the right MCA (arrow) and vascular blush of the meningioma (∗) (d).

**Figure 2:**
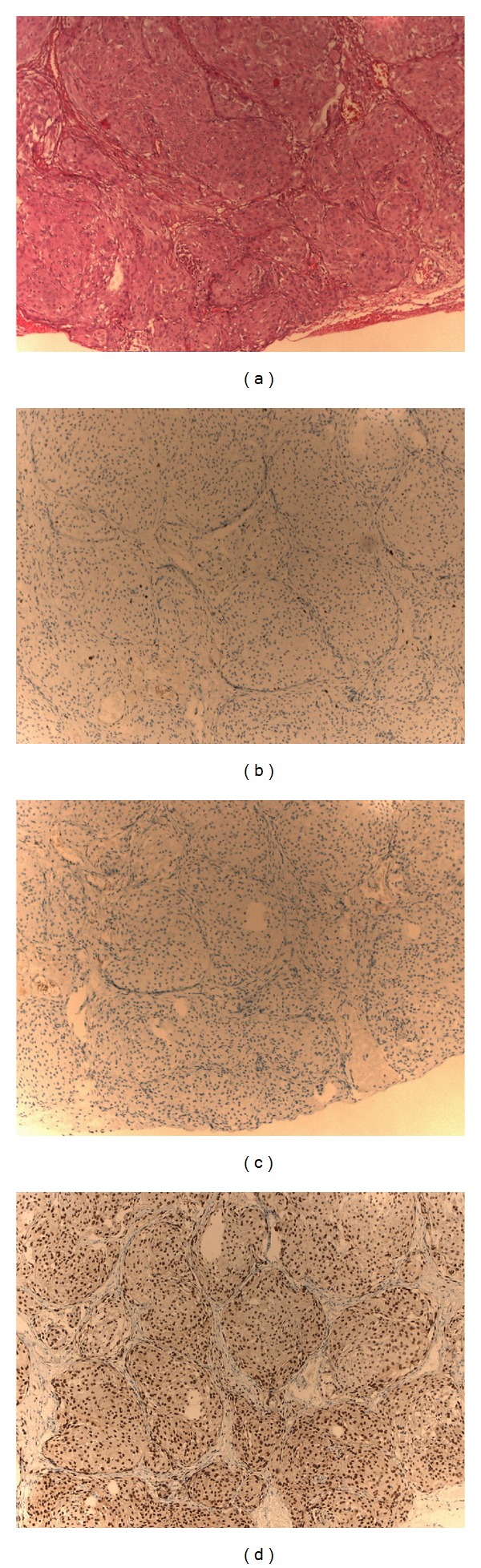
Meningothelial meningioma. Hematoxylin eosin staining section (original magnification ×100) showing characteristic cellular whorls (a). Immunohistochemistry (IHC) sections (original magnification ×100): the Ki 67 labelling index (1/100 *Dako*) indicates a low grade (1-2%) of the meningioma (b); IHC for estrogen receptors (*Ventana*) showing no positive nuclei (c), whereas IHC for progesterone receptors (*Ventana*) showing positive nuclei (d).

We report on a 30-year-old right-handed woman with a personal history of migraine admitted to the Department of Neurology, 1 month after childbirth, for acute onset of headache and complete paresis of her left hemibody, without sensory loss; National Institutes of Health Stroke Scale (NIHSS) was 10. Laboratory examination found no abnormality. T1-weighted magnetic resonance (MR) imaging with gadolinium disclosed a right sphenoid wing meningioma measuring 35 mm in diameter (Figure 1(a)). Diffusion-weighted MR imaging showed a hyperintense area in the right deep middle cerebral artery (MCA) territory (Figure 1(b)), indicating acute cerebral infarction; MR angiography sequences revealed interrupted arterial flow in the right MCA (Figure 1(c)). Selective angiography of the right carotid artery demonstrated an occlusion of the M1 portion of the right MCA and a rich vascular stain of the tumor, fed primarily by intracavernous branches of the right carotid siphon (Figure 1(d)). No cause for MCA infarct other than the meningioma was found. The tumor was totally removed. The MCA trunk was set in the meningioma, and a thrombus of the right MCA was observed during the surgery. The final pathological diagnosis confirmed a meningothelial meningioma with progesterone receptor positivity (Figure 2). No new neurological event occurred after surgery, and over the next several weeks, the patient recovered most of her neurological functions, with only a moderate spasticity of the upper and lower limbs. A brain MRI (six months later) showed no recurrence of meningioma.

## 3. Discussion

If encasement and intracranial arterial obstruction are common in cases of meningioma, strokes only rarely occur in association with this slowly growing tumor. In most of these cases, the different manifestations constitute a transient neurological deficit mimicking transient ischemic attacks probably due to hemodynamic stenoses. It has been suggested that meningiomal compression of the carotid artery may produce transient neurological symptoms including loss of consciousness, hemiparesis, paresthesia, and global transient amnesia [6]. However, complete cerebral infarction due to meningioma is exceedingly rare, and to our knowledge, only 6 other cases have been previously reported [7–10]: among these cases (including ours), 4 were MCA infarcts, 1 was an anterior cerebral artery infarct, 1 was a posterior watershed stroke, and 1 was a pontine infarct. Most of the patients were under 50 years of age. In our observation, neurological symptoms appear during postpartum period, only one month after childbirth. As is the case, progesterone receptors are expressed in 50 to 80% of human meningiomas [11]. It is well known that the response of meningioma to increased serum progesterone level during the second half of pregnancy may account for accelerated growth of this tumor [12], whereas a classical clinical and radiological regression of meningiomas during postpartum could be observed [13]. Pregnancy certainly played a crucial role in increasing the tumor volume of our patient.

A compressive mechanism is usually suggested as an explanation for this phenomenon. In fact, meningiomas involving the cavernous sinus commonly lead to encasement and narrowing of the internal carotid artery (8 to 100% of cases) [8], and invasion of the internal carotid artery by cavernous sinus meningioma is likewise possible but rare [14]. However, the incidence of meningioma-related cerebral ischemia by carotid artery compression is estimated at only 0.13% [6]. The reason why strokes are infrequent in this situation is still unknown, but it has been hypothesized that arteries (with high pressure) may be less compromised than cortical veins and dural sinuses (with low pressure) [6]. In conclusion, strokes due to meningioma are a highly rare clinical occurrence but should be given serious consideration, especially in young patients.
